# Collodion in Small Pox and Other Cutaneous Diseases

**Published:** 1851-12

**Authors:** 


*Collodion in Small Pox and other Cutaneous Diseases.* By E. McArthur, M. D., Chicago.

On the 20th of May last, I was called to visit Mr.——. I found him laboring under the usual symptoms peculiar to a severe attack of Bilious Fever. He was some 30 or more years of age, and possessed a strong and vigorous constitution. I inquired if he had been exposed to any person sick of Small Pox. He replied he had not. His symptoms being those peculiar to Fever of high arterial excitement, and at the same time his tongue being covered with a thick coat of brown fur, and there being much nausea and some vomiting, I ordered Calomel and Ipecac. aa grs. xvi M., to be given in powder. After a full half hour he commenced vomiting, at which time he was directed to take a little warm water occasionally, to facilitate and render vomiting as easy as might be. He threw up a large quantity of yellow and then copper-colored bile. I directed him to take Castor Oil ℥i. in four hours. His physic operated well and he took Dover powders and drank Cr. Tartar in solution, to promote perspiration and lessen fever. Next morning his pulse was very strong and quick, skin hot and dry, and no intermission of febrile symptoms. At evening his fever continued high and pulse strong, full and quick. There was a slight discoloration in each groin of the size of the palm of the hand. On the morning of the 22d I observed it was a case of Small Pox, and that the probability was that it would be one of a serious character. I hastily directed the application of Collodion to the whole face and neck. Four bottles of Collodion were used the next few days, it being applied every 4 hours. The patient remarked almost every time it was applied, "it feels so cool and good."

The disease progressed, and proved to be one of higher arterial excitement and severer in character than any I had ever seen, where the patient recovered, and I had treated several patients with the disease, each year for several years previously.

I have called attention to this case, not supposing that there was any thing peculiar or differing essentially from others, sufficient to interest the profession in the case itself. My object being to add the result of my experience to that of others, and thus to direct attention to the use of Collodion in diseases of this kind. My patient was pleased with its use, and I believe he was pitted far less than he would otherwise have been.

I have used the same remedy for different cutaneous diseases, in most of which, or all, it appeared to have a salutary effect by keeping the atmosphere from the part affected, and, at the same time, producing a cooling and agreeable sensation to the heated surface.

It has had a good effect in my practice in bad cases of cracked and otherwise sore nipples, and, if used early, for inflamed breasts of nursing women. *N. W. Med. and Surg. Jour.*

---

Dr. Farnham, convicted of conspiracy against the Michigan Central Railroad, and sentenced to the Penitentiary, is not a physician. His title arises from the fact that he has been practicing dentistry. The man whom the papers style Dr. Fitch, implicated in the same crime, was a farmer, and never either laid any claim to, or received the title of Doctor, until after the trial commenced. The only physician indicted was found innocent and set at liberty.—*N. W. Med. and Surg. Jour.*

